# The confounding effects of microvascular physiology on the uptake and diagnostic accuracy of [^18^F]fluoroethyl-tyrosine positron emission tomography in gliomas

**DOI:** 10.1007/s00259-026-07782-w

**Published:** 2026-02-21

**Authors:** Otto M. Henriksen, Thomas L. Andersen, Karine Madsen, Benedikte Hasselbalch, Dorte S. Nørøxe, Vibeke A. Larsen, Ulrich Lindberg, Henrik B. W. Larsson, Adam E. Hansen, Ian Law

**Affiliations:** 1 Dept. of Clinical Physiology Nuclear Medicine and PET, Copenhagen University Hospital Rigshospitalet, Copenhagen, Denmark; 2 Dept. of Clinical Medicine, Faculty of Health and Medical Science, University of Copenhagen, Copenhagen, Denmark; 3 Dept. of Oncology, Copenhagen University Hospital Rigshospitalet, Copenhagen, Denmark; 4 Danish Comprehensive Cancer Center. Brain Tumor Center, Copenhagen, Denmark; 5 Dept. of Radiology, Copenhagen University Hospital Rigshospitalet, Copenhagen, Denmark

**Keywords:** Glioma, Magnetic resonance imaging, Perfusion imaging, Positron emission tomography, Amino acid tracers

## Abstract

**Purpose:**

To investigate the physiological influence of tissue perfusion and permeability on O-(2-[^18^F]-fluoroethyl)-L-tyrosine ([^18^F]FET) uptake in gliomas by simultaneous MRI dynamic contrast enhanced (DCE) perfusion imaging and the impact of vascular contributions on diagnostic accuracy.

**Methods:**

Dynamic [^18^F]FET PET/MRI scans from 50 patients with glioma WHO grade 2–4 were analysed. In each patient multiple subvolumes were delineated to account for intra-patient heterogeneity. Associations of 20–40 min. [^18^F]FET tumour-to-background ratio (TBR_med_) with median blood volume (CBV), blood flow (F), and permeability (Ki) within 134 lesion subvolumes were investigated in linear mixed models. Also, associations with initial area under curve (iAUC_120_), time to peak (TTP) and slope (Slope_20 − 40_) from time activity curve were investigated. The influence of adjusting TBR_med_ for microvascular physiological parameters on the diagnostic accuracy for tumour recurrences was assessed in an independent dataset (*n* = 61 lesions from 48 patients).

**Results:**

CBV, F and Ki individually accounted for 42%, 36% and 26%, respectively, of the overall variance in TBR_med_. DCE metrics combined accounted for 49% of the total variance and for 65% of the regional variance in TBR_med_. All DCE metrics were associated positively with iAUC_120_ and negatively with both Slope_20 − 40_ and TTP (*p* < 0.001 for all). Adjusting TBR_med_ for microvascular effects lowered TBR by 24% on average and reduced the diagnostic accuracy (ROC AUC) from 0.90 to 0.77 in the test dataset.

**Conclusion:**

Microvascular properties may contribute to a considerable fraction of the clinical [^18^F]FET measures in patients with gliomas, and contribute positively to diagnostic performance of [^18^F]FET PET imaging.

**Supplementary Information:**

The online version contains supplementary material available at 10.1007/s00259-026-07782-w.

## Introduction

Positron emission tomography (PET) imaging using amino acid labelled tracers such as O-(2-[^18^F]-fluoroethyl)-L-tyrosine ([^18^F]FET), 3,4‑dihydroxy‑6‑[^18^F]‑fluoro-l‑phenylalanine (F-DOPA) or L-[methyl-^11^C]methionine ([^11^C]MET) are increasingly employed for glioma imaging as a supplement to conventional magnetic resonance imaging (MRI) [1]. Amino acid PET may in particular be useful for visualizing infiltrative tumour components [2] and for discriminating treatment related changes from tumour progression in the post-treatment setting [3].

Clinically used imaging metrics of tracer uptake based on tumour-to-background ratios (TBR) may be considered a simplified measure of the relative tracer distribution volume [4]. The exact mechanism of [^18^F]FET uptake in gliomas is still debated. Transport by L-amino acid transferase type 1 (LAT1) transporters is believed to be the main mediator of [^18^F]FET uptake in glioma tumour cells [5]. However, a study of IDH mutant gliomas showed no difference in LAT expression in [^18^F]FET negative and positive tumours according to standard TBR threshold [6]. Generally lower uptake in non-enhancing gliomas, and tracer accumulation also in non-neoplastic tissue, including vascular or inflammatory lesions [7], and the variable kinetics [8], further supports that other factors than specific LAT transport may influence tracer accumulation and consequently alter the association of TBR with tumour biology.

Several studies have reported measures of tumour perfusion, e.g. tumour blood volume (CBV) and blood flow (F), to be correlated with static [^11^C]MET and [^18^F]FET tumour activity [9–12]. As the two modalities reflect different aspects of tumour biology, the correlation may be considered to reflect common association with tumour malignancy. However, the strength of association has varied considerably among these comparative studies, and is likely depending on tumour characteristics, e.g. tumour type, blood-brain barrier (BBB) integrity, prior treatments including anti-angiogenic therapies, and methodological limitations in obtaining accurate physiological quantitation in MRI. Interestingly, some studies have shown that early uptake and washout may be closer associated with perfusion and vascular density, than with LAT expression and cellular density [13–15].

Thus, similarities and discrepancies between amino acid PET and CBV imaging may also reflect mechanisms of tracer accumulation. Indeed, uncertainties of the exact contribution of a disrupted BBB on passive radiotracer influx and tumour blood volume to tumour activity is a recurring theme in glioma imaging regardless of the tracers used [16–22]. Such non-specific activity due to leakage and circulating tracer in tumour vessels unrelated to LAT transport may underlie non-glioma tracer accumulation and potentially negatively influence diagnostic accuracy in patients with suspected recurrent glioma.

We have in our centre applied PET/MRI for routine imaging of treated glioma patients, particular for patients with suspected recurrent disease and patients scheduled for second line or experimental therapy. The protocol includes, in addition to [^18^F]FET PET and conventional MRI, also T1 weighted dynamic contrast enhanced (DCE) perfusion imaging that allows absolute quantification of both permeability (expressed as the influx transfer constant, Ki), blood flow (F) and blood volume (CBV) when applying a 2 compartment exchange model (2CXM) [23]. In prior analyses we have compared diagnostic performance [24] and prognostic value [25] of 2CXM DCE CBV and [^18^F]FET imaging in patients with high grade gliomas following standard therapy.

As a disrupted BBB may deliver an unascertained contribution to the [^18^F]FET uptake, the CBV may represent an indeterminate fraction [26], and as the activity in blood is variable compared to healthy brain, e.g. due to insufficient compliance to fasting [27], it is likely that the [^18^F]FET tumour metrics recommended for clinical use [28] and in trials [29] are systematically biased with possible detrimental effects on the diagnostic accuracy of the method. Thus, it is possible that improved diagnostic performance could be obtained by adjusting for these factors, or conversely that their contribution is overall positive. Different microvascular properties may also confound the observed associations between amino acid tracer uptake and molecular glioma subtype, e.g. for isocitrate dehydrogenase (IDH) wild-type vs. IDH mutant tumours [30, 31] as IDH wild-type glioma are associated both with higher uptake of [^18^F]FET and higher CBV [32].

The aims of the present analysis were to investigate contributions of quantitative estimates of vascular physiology derived from MRI DCE on established static and dynamic [^18^F]FET metrics across a wide spectrum of tumour biologies and imaging features, and to assess the impact of adjustment for these contributions on the diagnostic accuracy in separating tumour recurrence from treatment related effects in high grade glioma under identical physiological conditions using simultaneous acquisition with PET/MRI.

## Methods

### Patient population

We identified retrospectively 50 patients undergoing combined 40 min. dynamic [^18^F]FET PET and DCE MRI on our hybrid PET/MRI system between January 2016 and February 2021. Inclusion criteria comprised: (1) Adult patients (> 18 years) with treatment naïve or suspected recurrent/progressive previously treated low- or high-grade glioma (2) identifiable suspected tumour lesions on MRI or PET with one or more homogenous subvolumes of at least 0.5 cm^3^ in size, i.e. corresponding to volume of measurable lesions according to the RANO PET 1.0 [29] and MRI RANO 2.0 criteria [33]. Patients receiving antiangiogenic treatment or other non-standard therapy at time of scan, and scans with significant head motion were excluded.

Retrospective use of data was approved by the Danish Patient Safety Authority (reference no. 3–3013-1957/1) and for data from 2019 by the local hospital administration. A subset of the study population participated in a study approved by the local ethics committee (H1-2013-062) and conducted in accordance with the Helsinki Declaration, and participants gave informed written consent prior to the scan. The patient population partially overlaps with two prior publications comparing prognostic (*n* = 15) and diagnostic (*n* = 12) value of MRI DCE and [^18^F]FET PET in suspected recurrent high grade gliomas [24, 25].

### Imaging protocol

All imaging was performed on a Siemens Biograph mMR 3 T hybrid PET/MRI system equipped with a 16 channel head coil (Siemens Biograph, Siemens, Erlangen, Germany).

The hybrid PET/MR imaging protocol comprised a 40 min PET acquisition started at time of i.v. administration of approximately 200 MBq [^18^F]FET according to guidelines [28]. The static 20–40 min. image was reconstructed into a 344 × 344 matrix, voxel-size 0.8.x0.8 × 2 mm^3^, using 3D OP-OSEM (4 iterations, 21 subset) and applying a 5 mm Gaussian filter. The dynamic time series was reconstructed in to 14 frames (5 × 60 s, 5 × 180 s and 4 × 300 s) or 22 frames (6 × 10 s,4 × 15 s, 2 × 30 s, 2 × 60 s, 2 × 150 s and 4 × 300 s) applying a 172 × 172 matrix and a 3 mm filter, but otherwise same parameters. The attenuation correction was based on a separately obtained low-dose CT (120 kV, 30 mAs, 5 mm slice width, Siemens Biograph PET/CT system) as previously described [34] or a region-specific optimization of an UTE sequence (RESOLUTE) [35, 36]. Standard MRI included axial T2 BLADE (0.7 × 0.7 × 5 mm^3^), T2 FLAIR (1.2 × 0.9 × 5 mm^3^) and post-contrast 3D-T1 (1 × 1 × 1 mm^3^) weighted sequences.

DCE imaging was performed using a T1 weighted fast 3D spoiled gradient echo (VIBE) sequence with full brain coverage (30 slices, 2.4 × 2.4 × 5 mm^3^, TR/TE 2.94/0.86–0.91 s, flip angle 14°, linear phase encoding). T1-mapping were acquired before contrast injection using variable flip angles (4, 8, 14 and 20 or from Jan 2019 2, 3, 4, 6 and 8 degrees) but otherwise identical parameters. Dynamic imaging included 144 frames (temporal resolution of 2.55 s) acquired during a double bolus passage of 0.05 mmol/kg (Gadovist ^®^ 1 mmol/ml, Bayer, Berlin, Germany, injected at 18 and 85 s after the dynamic DCE acquisition was started) using a power injector (Medrad, Pittsburgh, PA) at a rate of 3 ml/sec followed by 10 ml of NaCl (3 ml/sec) [37, 38]. Processing of DCE data was performed using in-house software as previously described [23, 37, 39]. In short, blood flow (F) is estimated by model-free deconvolution with Tikhonov regularization, and subsequently CBV, Ki and the extra-vascular extra-cellular space (Ve) are fitted from the 2CXM kinetic analysis. As estimates of Ve is heavily biased by Ki (see also Suppl. Material, Table S1 and Fig. S1), Ve was not included in the analysis.

### Image analysis

The static and dynamic [^18^F]FET images, DCE parameter maps and structural MRI were co-registered and analysed using Mirada RTx software (Mirada Medical, Oxford, UK).

**Fig. 1:**
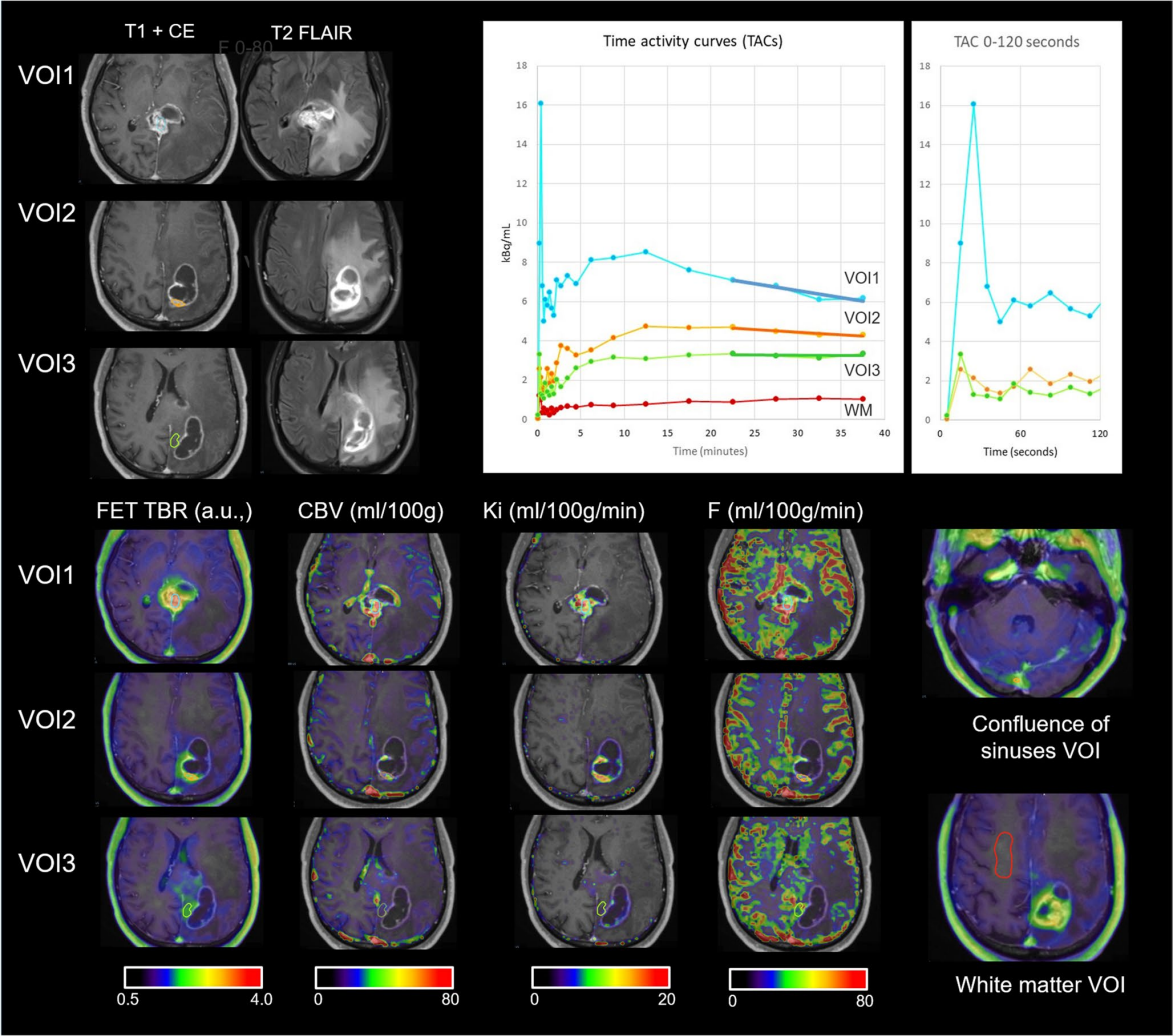
Example of VOI based analysis. Figure shows normal appearing white matter and lesion VOIs on MRI, static [^18^F]FET, DCE parameter maps and associated time activity curves (TACs) from a patient with multifocal recurrent glioblastoma. Linear fit of last part of the TAC used for calculation of normalized slope is shown, and first 120 s TAC for calculation of iAUC_120_ is presented in separate plot. Three lesion sub-VOIs representing high (VOI1, light blue) and moderately (VOI2, amber) active contrast enhancing, and non-enhancing (VOI3, light green) sub-VOIs

To account for intra-patient and/or intra-lesion heterogeneity, subvolumes were on the discretion of the investigator (OH) identified to obtain data from lesion components each with different imaging features on MRI (degree of enhancement) and PET (level of activity) and not necessarily representative of the patient or the whole lesion (Fig. 1). The number of subvolumes per patient thus depended on the number and heterogeneity of lesions. A spherical volume of interest (VOI) of at least 0.5 cm^3^ was placed within tumour components with homogenous appearance, while irregular shaped VOIs of similar size were manually delineated. Care was taken to minimize influence of vascular signals. In addition, a VOI of approx. 2 cm^3^ was drawn in normal appearing white matter (Fig. 1). From DCE maps median VOI values of F, Ki, CBV and Ve were obtained from each VOI. From static [^18^F]FET PET values of median and maximal activity within lesion VOIs were obtained to calculate median (TBR_med_) and maximal (TBR_max_) tumour-to-background ratio (i.e. relative to mean value from a cortical region in the contralateral hemisphere). To account for circulating tracer, the blood-to-background ratio (BBR) was calculated as the peak blood activity (mean of isocontour > 95% of maximum) within a region placed over the confluence of sinuses (Fig. 1) normalized by the mean background activity (same region as for calculation of TBR). For analysis of dynamic imaging the VOI was copied to each frame and the mean VOI value of each frame was obtained. Time to peak (TTP) and the slope of the last 20 min. of the TAC was determined by linear regression and normalized to peak activity (at TTP) to account for variable activity. Initial area under curve (iAUC_120_), expressed as standardised uptake value, of the first 120 s of the TAC was calculated.

### Tumour characterisation

From pathology reports prior to or within 1 month after imaging, tumour types were reclassified as astrocytoma, IDH mutant WHO grade 2–4, as oligodendroglioma 1p/19q co-deleted IDH mutant WHO grade 2–3, or glioblastoma IDH wildtype WHO grade 4 according to the 2021 WHO classification of glioma [40] (Table 1). Based on prior and follow-up imaging, histopathology and clinical decisions including multidisciplinary team conference, each patient was classified as having progressive (e.g. positive histopathology, clinico-radiographical progression or initiation of second-line therapy), or non-progressive disease (e.g. stable imaging, negative histopathology, or no change of treatment) within 6 months follow-up.

**Table 1:** Patient and lesion characteristics

	All	IDH wildtype*	IDH mutated	
		Glioblastoma	Astrocytoma	Oligodendroglioma
**Patients**,** n**	50	37	7	6
Progressive §, n (%)	44 (88)	33 (89.2)	6 (85.7)	5 (83.3)
WHO 2, n (%)	5 (10)	-	-	5 (83.3)
WHO 3, n (%)	5 (10)	-	4 (57.1)	1 (16.7)
WHO 4, n (%)	40 (80)	37 (100)	3 (42.9)	-
Days from last surgery/biopsy	179 [21–3095)	154 [21–1167]	480 [99–1590]	2422]104–3095]
Treatment naïve, n (%)	5 (10)	4 (10.8)	-	1 (16.7)
**Subvolumes**,** n**	134	95	20	19
Progressive,§ n (%)	121 (90.3)	87 (91.6)	18 (90)	16 (84.2)
Enhancing, n (%)	68 (50.8)	52 (55.6)	12 (60)	4 (21.1)

*One patient had Histon 3 mutated tumour, § from patients with progressive disease IDH = isocitrate dehydrogenase, WHO = world health organisation tumour grade

### Independent test dataset

Using the model parameters (as described later) determined in the present dataset, the influence of adjusting TBR_med_ for non-specific activity on diagnostic accuracy was investigated in a previously published dataset of patients with suspected recurrent high-grade gliomas undergoing [^18^F]FET PET/MRI DCE imaging [24]. Excluding data from 12 overlapping patients, a total of 61 lesions (33 progressive and 28 non progressive) from 48 unique patients were eligible for analysis (see also Suppl. Information). Diagnostic accuracy was investigated by receiver operating characteristics (ROC) analysis using 6 months follow-up as reference (*n* = 22 by histopathology) as previously described.

### Statistics

All values are reported as median (range) and groups compared using Mann-Whitney test. Due to skewed parameter distributions all DCE metrics, iAUC_120_ and TBR_med_ were analysed log2 transformed (of variable + 0.01 to include also values of 0) in regression and mixed model analysis. Associations of TBR_med_ and dynamic metrics with VOI derived median DCE values (CBV, Ki, and F) were initially investigated by scatter plots and crude linear regression with calculation of Pearson’s R^2^ (for TTP by Spearman’s correlation), and subsequently in 2-level linear mixed models to take into account multiple VOIs per patient. Each parameter of interest was entered as a fixed effect both in univariate and in multivariate models also including IDH status. For TBR_med_ as outcome BBR was also included. In the final multivariate model selection, a p-value criterion of 0.1 was applied. To assess effects on regional variability of TBR_med_ (ΔTBR_med_.), deviation of each VOI median or log2 median from mean of all VOI median or log2 median values were calculated and investigated in similar models not including the global fixed effects IDH status and BBR.

Non-specific [^18^F]FET activity in MRI lesions that could be attributed to leakage and circulating tracer in tumour vessels was estimated in a mixed linear model including (log transformed) values of Ki and tumour blood activity (TBA) calculated as

$$\:TBA=CBV\cdot\:BBR$$

Adjusted [^18^F]FET TBR_med_ (TBR_adj_) is then calculated from back-transformation of

$$\:Log2\:{TBR}_{adj}={Log2TBR}_{med}-\:{\beta\:}_{Ki}\cdot\:Log2\:Ki-\:{\beta\:}_{TBA}\cdot\:Log2\:TBA\:$$

where β_Ki_ and β_TBA_ denotes coefficients from a mixed linear model including only Ki and TBA. In the test dataset, whole lesion TBR_med_ was adjusted using β_Ki_ and β_TBA_ determined in the present analysis.

For mixed models marginal R^2^ (pesudo R^2^, pR^2^) was calculated by comparing sum of variance components of full model and a model without (level 1) predictors according to Snijders and Boskers method as implemented in STATA’s *mltrsq* function. Marginal R^2^ can similarly to R^2^ from Pearson’s correlation be considered a measure of the fraction or percentage (pR^2^*100%) of variance in the dependent variable (outcome) that can be explained by the model. Equality of the ROC area under the curves (AUCs) was performed using the DeLong test. All analyses were performed using STATA 15 (Stata Corp, College Station, Tx).

## Results

A total of 134 lesion VOIs were analysed. Patient and lesion characteristics are presented in Table 1. Five patients had biopsy/surgery within 30 (range 21–29) day before the scan. Five patients were treatment naïve and 45 patients had received prior therapies, of which two patients had been treated with bevacizumab (≥ 6 months) prior to the scan. White matter and lesion VOI summary statistics are provided in Table 2. All log-transformed DCE metrics were positively correlated (R^2^ 0.08–0.86, *p* ≤ 0.001 for all). Correlation matrix and scatterplots of selected pairwise correlations are provided in Suppl. material (Fig. S1 and Suppl. Table S1).

**Table 2:** Summary statistics by VOI type

	WM	Lesion SubVOIs	*p*-val	NCE	CE	*p*-val	IDH mut	IDH wt	*p*-val
	*n* = 50	*n* = 134		*n* = 65	*n* = 69		*n* = 39	*n* = 95	
TBR_med_ (a.u.)	0.70	2.15	< 0.001	1.93	2.51	< 0.001	1.96	2.21	0.401
TBR_max_ (a.u.)	0.92	2.50	< 0.001	2.23	2.87	< 0.001	2.29	2.51	0.651
TTP (minutes)	37.5	32.5	< 0.001	32.5	32.5	0.331	32.5	32.5	0.008
Slope_20 − 40_ (hour^− 1^)	0.523	0.139	< 0.001	0.168	0.068	0.046	0.317	0.064	< 0.001
iAUC_120_	0.012	0.033	< 0.001	0.027	0.044	< 0.001	0.026	0.037	0.017
F (ml/100 g/min)	8.41	20.53	< 0.001	14.02	24.37	< 0.001	15.36	22.40	0.002
Ki (ml/100 g/min)	< 0.01	1.23	< 0.001	0.00	6.10	< 0.001	0.19	1.78	0.012
CBV (ml/100 g)	0.72	2.29	< 0.001	1.52	4.50	< 0.001	1.78	2.61	0.020

Table shows median values. WM = white matter VOI = volume of interest, CE = contrast enhancing, NCE = non-enhancing, IDH mut = isocitrate dehydrogenase mutated, IDH wt = IDH wildtype, TBR = tumour-to-background ratio, TTP = time to peak, iAUC_120_ = initial area under curve, F = flow, Ki = influx transfer constant, CBV = cerebral blood volume

### Association with static [^18^F]FET PET

**Fig. 2:**
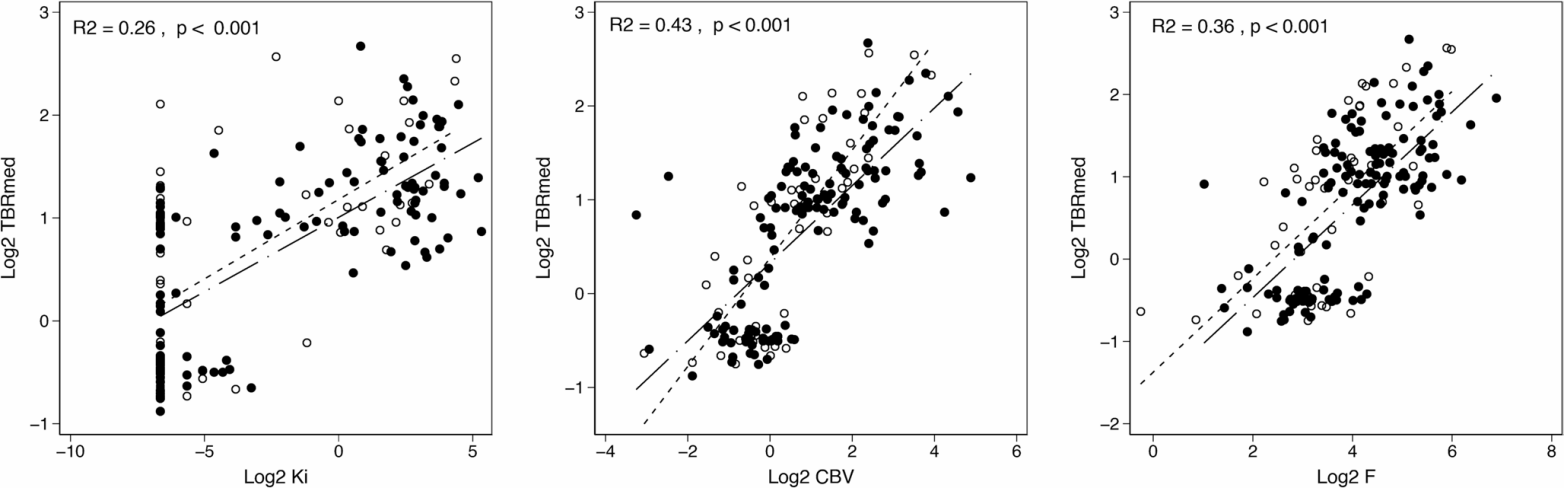
Association of DCE metrics with median [^18^F]FET TBR. Separate regression lines are shown for VOIs from patients with IDH wildtype (solid with long dashed line) and IDH mutant (hollow with short dashed line) tumours. The association of TBRmed with CBV was stronger (β = 0.471 vs. 0.239, *p* < 0.001 for interaction) in subvolumes from patients with for IDH mutant (R^2^ = 0.63, *p* < 0.001) than IDH wildtype (R^2^ = 0.36, *p* < 0.001) tumours. Otherwise, no significant interactions with IDH status were observed in univariate analysis. Overall R^2^ and p-value are shown

Scatterplots with associations of VOI metrics with TBR_med_ are shown if Fig. 2 showing stronger association of TBR_med_ with CBV in IDH mutant tumours. No significant interactions with contrast enhancement could be demonstrated, although the effect of Ki was only significant in non-enhancing (R^2^ = 0.10, *p* = 0.008) and not in enhancing (R^2^ = 0.02, *p* = 0.271) subvolumes. In univariate mixed models (Table 3) TBR_med_ was positively associated with increasing Ki, F, and CBV, where CBV and F showed the strongest association (marginal R^2^ of 0.42 and 0.36, respectively).

**Table 3:** Mixed linear model analysis – univariate models

	All (*n* = 134)				IDH wt (*n* = 95)				IDH mut (*n* = 39)			
	TBR_med_		ΔTBR_med_ †		TBR_med_		ΔTBR_med_ †		TBR_med_		ΔTBR_med_ †	
	Coeff.	pR^2^	Coeff.	pR^2^	Coeff.	pR^2^	Coeff.	pR^2^	Coeff.	pR^2^	Coeff.	pR^2^
Log2 CBV	0.301***	0.421	0.327***	0.524	0.253***	0.357	0.286***	0.520	0.469***	0.630	0.452***	0.590
Log2 F	0.394***	0.357	0.457***	0.453	0.338***	0.333	0.369***	0.382	0.626***	0.403	0.717***	0.668
Log2 Ki	0.0835***	0.256	0.101***	0.328	0.075***	0.264	0.085***	0.308	0.120***	0.240	0.153***	0.418
Log2 BBR	0.118	0.004	.	.	0.041	0.001	.	.	0.235	0.018	.	.
IDH mut	0.0860	.	.	.	.	.	.	.	.	.	.	.

* *p* < 0.05, ** *p* < 0.01, *** *p* < 0.001,†mean subtracted fixed effect, pR^2^ = marginal R^2^, IDH mut = isocitrate dehydrogenase mutated, IDH wt = IDH wildtype, BBR = blood-to-background ratio, F = flow, Ki = influx transfer constant, CBV = cerebral blood volume

In multivariate models (Table 4) DCE parameters (CBV, F and Ki) accounted for 49% of the total variance in TBR_med_.

**Table 4:** Mixed linear model analysis – multivariate models

	All (*n* = 134)			IDH wt		IDH mut	
	TBR_med_		ΔTBR _med_†	TBR_med_	ΔTBR _med_†	TBR_med_	ΔTBR _med_†
	DCE + IDH	DCE only					
Log2 CBV	0.153***	0.155***	0.149***	0.018**	0.154***	0.308**	0.039
Log2 F	0.167**	0.215***	0.262***	0.203***	0.207***	0.175	0.534***
Log2 Ki	0.0430***	0.0334**	0.047***	0.036**	0.038***	0.033	0.040***
IDH mut	−0.229*	.	.	.	.	.	.
pR^2^	0.534	0.485	0.649	0.473	0.633	0.624	0.738

* *p* < 0.05, ** *p* < 0.01, *** *p* < 0.001, †mean subtracted fixed effect, pR^2^ = marginal R^2^, IDH mut = isocitrate dehydrogenase mutated, IDH wt = IDH wildtype, F = flow, Ki = influx transfer constant, CBV = cerebral blood volume

Similar, and slightly stronger effects were observed in analysis of regional deviations (see also Suppl. Fig. S2), where regional variation DCE metrics combined accounted for 65% of regional variance in ΔTBR_med_.

### Time-activity-curve

**Fig. 3:**
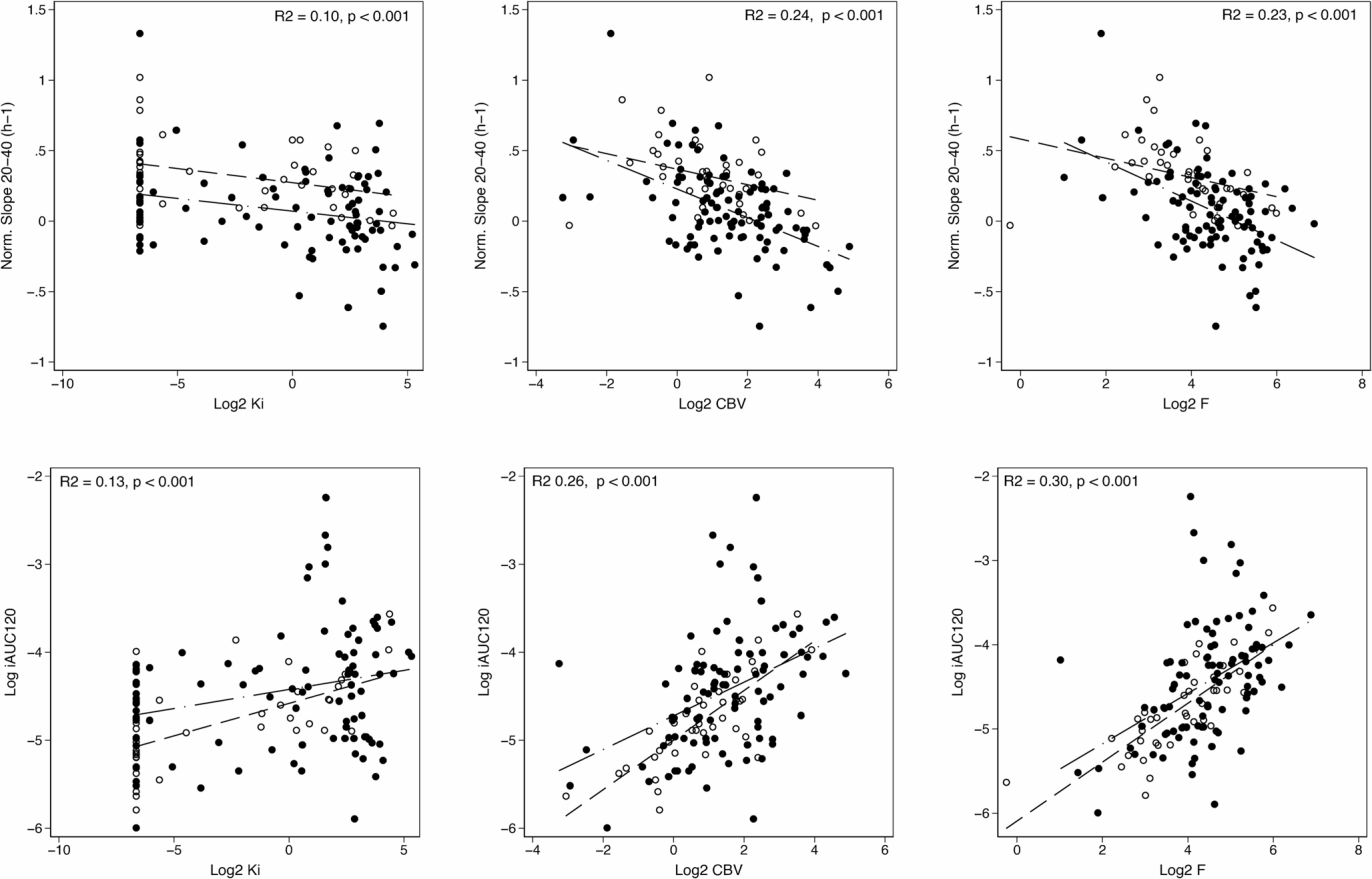
Association of DCE metrics with normalised slope and initial area under curve (iAUC_120_) from [^18^F]FET time-activity curve. Separate regression lines are shown for VOIs from patients with IDH wildtype (solid with long dashed line) and IDH mutant (hollow with short dashed line) tumours. As no significant interaction with IDH status was found for any correlations, only overall R^2^ and p-value are shown

Both CBV, F and Ki were associated positively with iAUC_120_ and negatively with Slope_20 − 40_ (*p* < 0.001 in univariate analysis, Fig. 3 and Suppl Table S2), The association was weaker for Ki that did not show statistically significant effects in multivariate mixed models, where F and CBV combined accounted for 29% of variance in Slope_20 − 40_ and 36% of the variance in iAUC_120_ (Suppl. Table S3). For TTP a highly significant negative associations with CBV (Spearman’s rho − 0.33, *p* < 0.001) and F (Spearman’s rho − 0.28, *p* = 0.001) were observed while association with Ki (Spearman’s rho − 0.20, *p* = 0.02) was weaker. In ad hoc mixed model analysis iAUC_120_ accounted for 39% of variance in TBR_med_, for 77% of regional variance in TBR_med_.

### Adjusted TBR

**Fig. 4:**
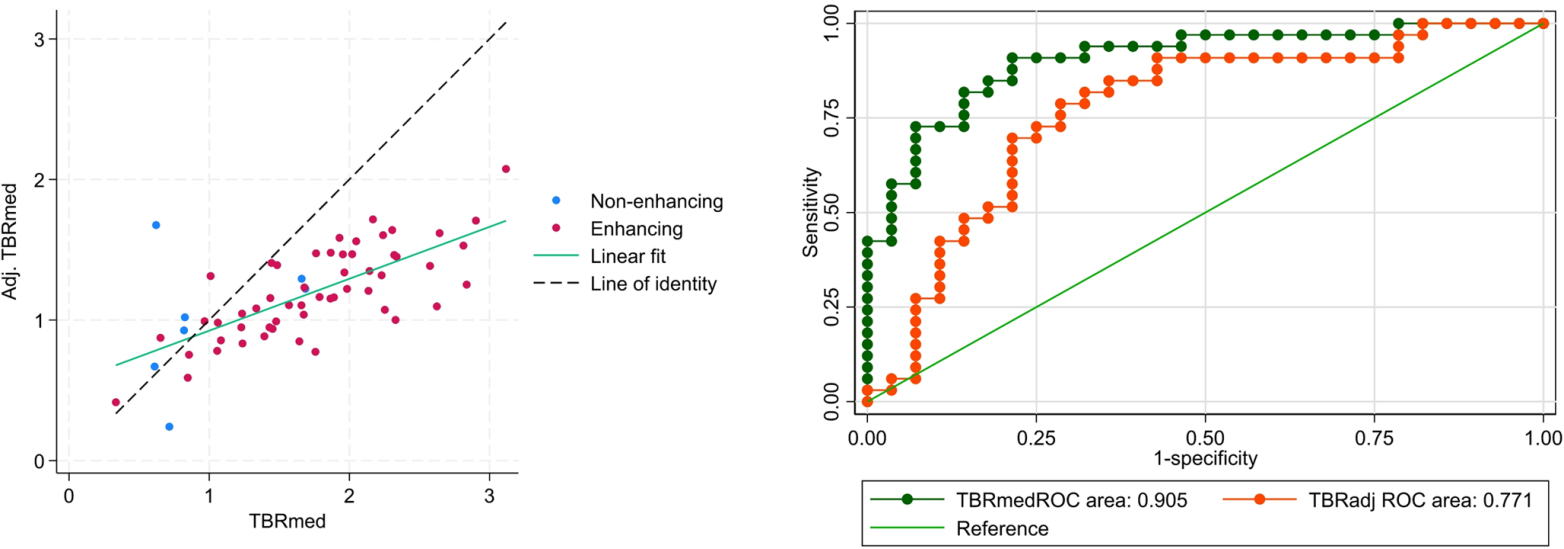
Adjustment of TBR_med_ for non-specific activity in the independent test dataset. Left scatterplots of TBR_med_ vs. TBR_adj_. and right corresponding ROC curves

Adjusting for Ki and TBA effects, TBR_med_ was reduced on average by 24% both in the current data set and in the independent test dataset with diagnostic outcome. ROC analysis showed significantly lower ROC AUC of TBR_adj_ compared to unadjusted TBR_med_ (ROC AUC 0.77 vs. 0.90, *p* = 0.007). Scatter plot of adjusted vs. unadjusted TBR and ROC curves are provided in Fig. 4.

## Discussion

In the present study we investigated the associations of DCE perfusion parameters with tracer accumulation. To our knowledge, no previous studies have analysed the possible direct contribution of quantitative DCE MRI measurements of permeability, perfusion and blood volume on static and dynamic [^18^F]FET metrics in humans. The results show that static [^18^F]FET PET uptake was positively associated with measures of perfusion and permeability, and that microvascular function and physiology characterized by DCE contributed considerably to tracer uptake variability accounting for half of the overall and two thirds of the regional variance in static tracer uptake. Importantly, adjusting for the vascular contribution to tracer accumulation *lowered* the accuracy in suspected recurrent glioma indicating a significant positive contribution of vascular physiology in the diagnostic performance of [^18^F]FET PET. Finally, the analysis supported and association of microvascular function with TAC derived measures of rate of tracer accumulation and wash-out.

Among DCE metrics CBV and F had the largest effect on static and dynamic PET metrics. Previous studies have mainly focused on the association of amino acid tracer uptake with CBV measured by DSC more widely applied in clinical perfusion imaging in gliomas [9, 11, 12, 41]. As opposed to DSC, DCE allows absolute quantification of both CBV, blood flow (F) and permeability (Ki). In the present analysis we also included values of Ki and F within VOIs analysed, and found both Ki, F and CBV to be correlated with [^18^F]FET uptake, also in multi-variate models. This finding is opposed to a previous published study that relied on a ratio between pre- and post-contrast T1-weighted MRI as surrogate marker for BBB leakage [42] indicating oversimplification of model assumptions as the post-contrast T1-weighted MRI is a non-calibrated signal and a composite of Ki, F and CBV at any given time. Thus, the present study demonstrates the added value of increasing the physiological accuracy. As we did not have pre-contrast enhanced 3D T1 MRI on all patients we were not able to compare DCE with such simpler approaches.

The observed associations of CBV and [^18^F]FET uptake are in agreement with previous human histopathological studies. One study showed an association of both DSC CBV and [^11^C]MET with endothelial proliferation in gliomas [43] and recently another study reported an association of both static and dynamic (washout) [^18^F]FET uptake metrics with vascularization in treatment naïve glioblastomas [13]. Of particular interest we found an association of permeability with [^18^F]FET uptake, most pronounced in visually non-enhancing VOIs indicating a permissive role of only mildly increased permeability. This contrasts with the findings of animal studies which concluded no substantial influence on [^18^F]FET uptake when restoring BBB permeability by dexamethasone [44] or bevacizumab [45]. Thus, although BBB disruption may contribute to the uptake it is not a prerequisite for intra-tumoural accumulation. Anti-angiogenic treatments could potentially influence the association of microvascular properties and tracer accumulation.Two patients had previous exposure to bevacizumab more than 6 months prior to the scan. Both were IDH mutant and median lesion VOI values did not differ from other patients with IDH mutant tumours for any [^18^F]FET or DCE parameter, and therefor unlikely to have confounded the analysis.

Blood flow quantified by regularized model-free convolution showed moderate correlation with CBV and static [^18^F]FET uptake. A relatively close correlation of F and CBV is expected as CBV and F are related by the mean transit time (F = CBV/MTT). Furthermore, F measured by DCE reflects intravascular flow and not perfusion as measured by a freely diffusible tracer such as [^15^O]H_2_O, and, thus, is more dependent on intravascular volume. Still, the positive association of F and [^18^F]FET uptake is in accordance with overall findings in two previous published smaller studies measuring blood flow by [^15^O]H_2_O [4, 46].

Association of DCE parameters with TBR_med_ tended to be stronger in IDH mutant compared to IDH wildtype tumours, possible owing to larger variability in the lower range of Ki, CBV and Ki, but appears to follow an overall common association, also for dynamic parameters, and suggests an underlying class related effect of IDH status. Thus, the lower tracer uptake and increasing TAC more often found in IDH mutant tumours compared to IDH wildtype tumours [30, 31] may at least partially reflect differences in microvascular properties [32]. Still, some tumours such as oligodendroglioma may appear highly metabolic active despite relatively slow growth and both preserved BBB and low vascularity, and thus clearly demonstrating that other tumour specific properties contribute to tracer accumulation.

The physiological basis of the early peak and decreasing TAC slope typically associated with higher tumour grades and IDH wildtype status has not been established. From our data, the associations of Slope_20 − 40_ with CBV and F appeared stronger than that with leakage (Ki). Together vascular factors accounted for less than 30% of the variance in Slope_20 − 40_, and the basis of the residual larger fraction of variance remains unaccounted for. As the amino-acid analogue [^18^F]FET is not believed be utilized for protein synthesis, the concept of unidirectional accumulation of tracer in glioma cells may be an oversimplification, and efflux from the cells by a yet unknown mechanism could contribute decreasing TAC.

As first pass extraction of FET is low when blood brain barrier is intact [4, 47] and magnitude of LAT mediated uptake within the first 2 minutes is probably minimal, the early peak in TAC can predominantly be attributed to intravascular circulating tracer and to some extent leakage. Our analysis of initial area under curve (iAUC_120_) showed fair agreement with tumour perfusion as reflected by CBV and F, and showed similar associations with TBR_med_. For regional deviations in TBR_med_, the iAUC_120_ in fact yielded the highest marginal R^2^ of 0.77 in univariate analysis, thus outperforming DCE derived metrics. The findings are partially in agreement with a recent study reporting that early TBR from first 2 minutes was strongly correlated with CBV measured by DSC (R^2^ = 0.79), while correlation with late TBR (20–40 min) in that study did not reach statistical significance (R^2^ = 0.08, *p* = 0.11) [48].

Adjusting lesion activity for the effects of leakage and circulating tracer in intra-tumoural vessels reduced TBR_med_ by 24% on average. This lower adjusted measure (TBR_adj_) could be considered a more specific measure of LAT dependent tracer uptake and could potentially improve diagnostic accuracy, particularly in the presence of treatment related effects that may also exhibit increased permeability and tracer uptake. However, when applied to an independent test set, diagnostic accuracy was significantly lower. This observation could suggest that non-LAT mediated tracer uptake related to vascular factors substantially contributes to the high diagnostic accuracy of [^18^F]FET imaging, and explain why adding CBV imaging to amino acid PET results in no or only minimal gain in diagnostic accuracy [24, 49–51].

Although these observations may point to an influence of microvascular function on tracer uptake, it could also reflect confounding factors due to underlying variation in tumour malignancy. In a biopsy control study of treatment-naïve glioblastomas, [^18^F]FET TBR correlated with both LAT1 tissue immunoreactivity and CD31-positivity, a vascularization marker [13] Thus, the TBR value may reflect both the expression of LAT1 in tissue as well as parameters related to neoangiogenesis such as increased flow, blood volume and permeability with passive tracer influx across a disrupted BBB as suggested by the present findings. It should be noted that the Ki is measured for the gadolinium contrast agent, not for [^18^F]FET. By inference any BBB disruption that would allow the much bulkier contrast agent to passively pass through would also allow the same for [^18^F]FET that have only 37% of the molecular weight. Thus, the estimated 0.03–0.08 Log2 TBR_med_ units per doubling in Ki (corresponding to 15–41% TBR_med_ increase in enhancing lesion vs. non-enhancing lesions) should thus be understood as a minimum average contribution. Establishing Ki as a surrogate marker of [^18^F]FET permeability would require a formal full kinetic compartment modelling with 90 min. acquisition and arterial plasma input function as has only been performed in the above mentioned study of seven untreated glioma patients so far [4]. In three glioblastomas the rate constants from plasma to tissue (K1) were 2 to 20 ml/100 g/min, and within range of our values measuring up to 40 ml/100 g/min.

The present analysis has some important limitations. First, the study design can only demonstrate associations in linear models, but not establish causality. Due to the diversity of prior treatments, the potential confounding effects of specific treatments or combinations of treatments could not be investigated. Also, only simplified static and dynamic PET metrics were applied. In that aspect the introduction of long axis field of view scanners allowing for both dynamic imaging with high temporal resolution and simultaneous measurement of image derived input function from the aorta [47] may facilitate kinetic analysis of PET data and greatly increase our understanding of tracer kinetics in brain tumours. The included patients constituted a convenience sample of individuals who underwent both dynamic FET and DCE that in our centre is mainly applied in research setting or for selected cases. Also, lesion VOIs were relatively small to analyse homogenous tumour components, which may increase influence of partial volume effects (due to differences in resolution), and motion related effects. Finally, VOIs were selected to increase heterogeneity of data and, thus, not necessarily representative of the whole tumour volumes which may have biased results. Still, results appear robust and do no suggest that overall findings to be the result of the above-mentioned limitations.

## Conclusions

The present analysis shows that accumulation of [^18^F]FET in glioma WHO grade 2–4 may be influenced by increased permeability, blood volume and perfusion with an average collective contribution of nearly 50%, but shows also that these effects may independently contribute to the high diagnostic value of [^18^F]FET PET imaging with adjustments of these factors being unwarranted.

## Supplementary Information

Below is the link to the electronic supplementary material.

Supplementary File 1 (DOCX 185 KB)

## Data Availability

Data used and/or analysed during the current study are available from the corresponding author on reasonable request.
