# Supplementary material for: The confounding effects of microvascular physiology on the uptake and diagnostic accuracy of [18F]fluoroethyl-tyrosine positron emission tomography in gliomas
Source: Eur J Nucl Med Mol Imaging. 2026 Feb 21;53(6):4025–36. doi: 10.1007/s00259-026-07782-w (PMC13121211; doi:10.1007/s00259-026-07782-w)
Supplement: Supplementary file 1 — (DOCX 185 KB) [file 259_2026_7782_MOESM1_ESM.docx]

**Suppl. Information**

**Title**

**The confounding effects of microvascular physiology on the uptake and diagnostic accuracy of [^18^F]fluoroethyl-tyrosine positron emission tomography in gliomas.**

Authors

Otto M. Henriksen^1^, Thomas L. Andersen^1,2^, Karine Madsen^1^, Benedikte Hasselbalch^3,4^, Dorte S. Nørøxe^3,4^, Vibeke A. Larsen^5^, Ulrich Lindberg^1^, Henrik B.W. Larsson^1,2^, Adam E. Hansen^2,4,5^, Ian Law^1,2^

^1^ Dept. of Clinical Physiology Nuclear Medicine and PET, Copenhagen University Hospital Rigshospitalet, Copenhagen, Denmark

^2^ Dept. of Clinical Medicine, Faculty of Health and Medical Science, University of Copenhagen, Copenhagen, Denmark

^3^ Dept. of Oncology, Copenhagen University Hospital Rigshospitalet, Copenhagen, Denmark

^4^ Danish Comprehensive Cancer Center. Brain Tumor Center, Copenhagen, Denmark

^5^ Dept. of Radiology, Copenhagen University Hospital Rigshospitalet, Copenhagen, Denmark

**Independent dataset**

The original dataset set comprised 76 lesions from 60 unique patients analysed to determine the diagnostic accuracy of DCE blood volume for differentiation of tumour progression vs treatment effects in high-grade gliomas. Imaging was performed due to variable suspicion of tumour recurrence following standard therapies only, and included [^18^F]FET PET imaging and MRI protocol similar that applied in the current study. All patients had diffuse astrocytoma IDH mutant WHO grade 3-4 (n=10 patients with 12 lesions) or glioblastoma IDH wildtype WHO grade 4 (n=38 patients with 49 lesions) when reclassified according to the WHO 2021 criteria.

**Suppl Table S1. Correlation matrix of DCE and PET metrics**

|  | Log2 Ve | Log2 CBV | Log2 Ki | Log2 F | TBRmed | Log2 iAUC_120_ | Slope_20-40_ |
| --- | --- | --- | --- | --- | --- | --- | --- |
| Log2 Ve | - |  |  |  |  |  |  |
| Log2 CBV | 0.5651* | - |  |  |  |  |  |
| Log2 Ki | 0.9282* | 0.5955* | - |  |  |  |  |
| Log2 F | 0.4671* | 0.6506* | 0.4231* | - |  |  |  |
| TBRmed | 0.4974* | 0.6546* | 0.5088* | 0.5986* | - |  |  |
| Log2 iAUC_120_ | 0.3751* | 0.5085* | 0.3637* | 0.5496* | 0.6678* | - |  |
| Slope_20-40_ | -0.3087* | -0.4933* | -0.3212* | -0.4827* | -0.4306* | -0.4741* | - |

*p<0.05

**Suppl. Table S2. Mixed linear model analysis – dynamic metrics**

|  |  |  | **Slope_20-40_** |  | **iAUC** |  |
| --- | --- | --- | --- | --- | --- | --- |
|  |  |  | Coeff. | pR2 | Coeff. | pR2 |
| Univariate | Log2 CBV |  | -0.1000*** | 0.241 | 0.222*** | 0.273 |
|  | Log2 F |  | -0.133*** | 0.230 | 0.318*** | 0.320 |
|  | Log2 Ki |  | -0.023*** | 0.101 | 0.055*** | 0.125 |
|  | IDH mut |  | -0.241*** | 0.126 | -0.271 | 0.04 |
| Multivariate | CBV+F |  |  | 0.286 |  | 0.358 |
|  | CBV+F+IDH |  |  | 0.343 |  |  |

* p<0.05, ** p<0.01, *** p<0.001, pR2 marginal R2, †mean subtracted fixed effect

**Figure S1. Scatter plots of pairwise correlations between DCE metrics.** For crude linear correlation coefficients, see Suppl. Table S1.

**Figure S2. Scatterplots of regional deviations in TBR_med_, and DCE metrics.** Regional deviation is calculated deviation from mean of subvolumes in each patient.
